# Increased HMGB1 and cleaved caspase-3 stimulate the proliferation of tumor cells and are correlated with the poor prognosis in colorectal cancer

**DOI:** 10.1186/s13046-015-0166-1

**Published:** 2015-05-20

**Authors:** Zhengxiang Zhang, Min Wang, Ling Zhou, Xiao Feng, Jin Cheng, Yang Yu, Yanping Gong, Ying Zhu, Chuanyuan Li, Ling Tian, Qian Huang

**Affiliations:** The Comprehensive Cancer Center & Shanghai Key Laboratory for Pancreatic Diseases, Shanghai General Hospital, School of Medicine, Shanghai Jiao Tong University, Shanghai, 201620 China; The Department of Surgery, The Branch Hospital of Shanghai General Hospital, Shanghai, 200080 China; Experimental Research Center, Shanghai General Hospital, School of Medicine, Shanghai Jiao Tong University, Shanghai, 201620 China; Department of Dermatology, Medical Center, Duke University, Durham, NC 27710 USA

**Keywords:** Colorectal cancer, Caspase-3, High mobility group box 1(HMGB1), Dying cells, Tumor cell proliferation

## Abstract

**Background:**

Dying tumor cells after irradiation could promote the proliferation of living tumor cells might cause tumor relapse and treatment failure. Our previous study showed that activated caspase-3 after irradiation probably participates in tumor repopulation. In this study, we investigated whether high mobility group box 1(HMGB1) is also involved in tumor repopulation.

**Methods:**

Colorectal tumor cells were irradiated. The cleaved caspase-3 (CC3) in irradiated tumor cells and HMGB1 in the supernatant of irradiated tumor cells were detected by Western blot. A large number of irradiated colorectal tumor cells (feeder cells) were then co-cultured with a small number of luciferase-labeled living colorectal tumor cells (reporter cells) and proliferation of reporter cells was measured by bioluminescence imaging. The CC3 and HMGB1 protein expression in colorectal tumor and peritumoral tissues were detected by immunohistochemistry and their correlation with prognosis were analyzed.

**Results:**

The irradiated colorectal tumor cells underwent apoptosis and necrosis and produced CC3 in tumor cells and HMGB1 in the supernatant of cultured cells. The increased expression of secretory HMGB1 correlated with CC3 level and proliferating cell nuclear antigen (PCNA) after irradiation *in vitro*. The irradiated dying cells remarkably stimulated living tumor cell proliferation. Interestedly, immunohistochemistry staining showed that positive HMGB1, CC3, and Ki67 expression were significantly higher in colorectal tumor tissues than in peritumoral tissues (*p* <0.01). The Kaplan-Meier survival analysis revealed that high HMGB1, CC3, and Ki67 levels were significantly associated with poor prognosis (*p* <0.05, *p* <0.01). Multivariate analysis using Cox proportional hazards model showed that TNM staging and HMGB1 were independent prognostic factors in patients with colorectal cancer (CRC) (*p* <0.01, *p* <0.001).

**Conclusion:**

Both apoptotic and necrotic cells could stimulate proliferation of living tumor cells, and the increased expression of CC3 and HMGB1 in tumor cells could be new markers for poor prognosis in colorectal cancer patients.

**Electronic supplementary material:**

The online version of this article (doi:10.1186/s13046-015-0166-1) contains supplementary material, which is available to authorized users.

## Introduction

Resistance to chemotherapy and radiotherapy is a universal obstacle in cancer treatment and one of the main causes of tumor relapse and treatment failure. However, the mechanisms responsible for drug or radiation resistance have yet to be fully elucidated [1]. A recent study of ours demonstrated that dying tumor cells after radiotherapy can stimulate the proliferation of residual living tumor cells, which may be a possible mechanism of tumor resistance and repopulation after radiotherapy [2]. This process was found to be associated with the activation of caspase-3 and caspase-7. The activated caspases can further activate downstream effectors. One of these key effectors is cytosolic calcium-independent phospholipase A_2_ (iPLA_2_), which can promote prostaglandin E_2_ (PGE_2_) production. PGE_2_ can then stimulate the proliferation of living tumor cells *in vitro* and *in vivo* [2, 3]. A recent study by Kurtova et al. also demonstrated that chemotherapy effectively induces apoptosis and PGE_2_ release, which paradoxically promotes neighboring cancer stem cell repopulation and leads to subsequent chemoresistance [4]. Ford et al. also demonstrated that apoptotic tumor cells promoted tumor growth, angiogenesis, and accumulation of tumor associated macrophages in aggressive B cell lymphomas [5]. However, tumor resistance and progression after therapy is a highly complex process and many signaling pathways may be involved, and whether cell death signaling pathways other than apoptosis could promote tumor progression has not been clarified.

High mobility group box 1 (HMGB1) is a highly conserved nuclear protein. In cell nuclei, HMGB1 functions as a DNA chaperone to regulate DNA replication, recombination, transcription, and repair [6]. Also, HMGB1 can be released or secreted from cells into the extracellular matrix to function as a signaling molecule. In general, HMGB1 is passively released from dead, dying, or injured cells. Therefore, extracellular HMGB1 is thought to be an optimal “necrosis marker” selected by the innate immune system to recognize tissue damage [7]. The extracellular HMGB1 binds to several cell surface receptors, including the receptor for advanced glycation end products (RAGE) [8]. RAGE was the first receptor demonstrated to bind HMGB1 [9], and the binding of HMGB1 to RAGE stimulates proliferation and differentiation of cancer cells as well as tissue regeneration [10]. Recent studies showed that HMGB1 could promote chemotherapy resistance in colorectal cancer and lung adenocarcinoma through HMGB1 mediated autophagy [11, 12]. In the clinic, the expression of RAGE is closely associated with invasion and metastasis of gastric cancer [13] and colorectal cancer [14]. Hongo et al. also found that HMGB1 expression detected by immunohistochemistry correlates with the resistance of preoperative chemoradiotherapy in lower rectal cancer [15].

In this study, the role of HMGB1 in CRC tumor cell proliferation induced by irradiated dying cells was investigated *in vitro*, and the clinical significance of HMGB1 expression in colorectal cancer patients was analyzed by comparing its expression with the expression of CC3, RAGE, and Ki67.

## Materials and methods

### Cell culture and irradiation

HT29, HCT8, SW620, CaCO2, RKO cells were purchased from the Chinese Academy of Science (Shanghai, China) and cultured in Dulbecco’s Modified Eagle’s Medium (DMEM) (Thermo Scientific Inc., Beijing, China) containing 10 % fetal bovine serum (FBS) (Tianhang Biological Technology Co., Ltd., Hangzhou, China), supplemented with 1 % penicillin and 1 % streptomycin at 37 °C under 5 % CO_2_. Cells were irradiated with X-rays using an Oncor linear accelerator (Siemens, Amberg, Germany). The dose rate was approximately 3.6 Gy/min.

### Tumor cell proliferation model *in vitro*

HT29 cells irradiated with various doses of X-rays were seeded into 24-well plates at a density of 2.5 × 10^5^/well. After the cells became attached (about 6 h), the reporter cells (non-irradiated HT29 with firefly luciferase and GFP fusion gene, Fluc), were seeded into the plates at a density of 1000/well and co-cultured for about 2 weeks. The substrate D-luciferin (Promega, Madison, WI, USA) prepared for a final concentration of 0.15 mg/ml was added, and the luciferase activity was detected with NC100 instrument (Berthold Technologies, Bad Wildbad, Germany). The final luciferase signal readouts were analyzed quantitatively by the manufacturer supplied software and represented the status of accelerated proliferation of reporter cells.

### Western blot

Total protein was extracted with RIPA lysis buffer containing protease inhibitor cocktail (Roche). Western blot was performed as previously described [16]. The membranes were incubated with primary antibodies purchased from Cell Signaling Technology (MA, USA. HMGB1, 1:1000 dilution; PCNA, 1:1000 dilution; CC3, 1:1000 dilution; β-actin, 1:1000 dilution) overnight at 4 °C and then incubated with secondary antibodies (1:5000 dilution, Jackson Immuno Research, PA, USA) for 2 h at room temperature. ECL Plus (Roche, Basel, Switzerland) was used to visualize the signals on the membrane.

To detect secretory HMGB1, cell culture supernatants were collected at 0 h, 6 h, 12 h, 1 day, 2 day, 3 day, 4 day, and 5 day. Secretory HMGB1 was detected through Western blotting shown above. Gray value of the bands in the Western blot was analyzed using ImageJ software [17].

### Flow cytometry of cell death

Exponentially growing HT29 cells were seeded into 6-cm dishes and irradiated. Cells were trypsinized and suspended 48 h after irradiation. The suspended cells were stained with Annexin V and propidium iodide (PI) using FITC Annexin V Apoptosis Detection Kit (BD company, USA) and subjected to flow cytometry using Accuri C6 Flow Cytometer (BD company). AnnexinV^+^ PI^−^ cells were regarded as apoptotic cells, while AnnexinV^+^ PI^+^ cells as necrotic cells [18, 19].

### Patient selection

A total of 73 patients that underwent radical resection for CRC between January 1, 2009 and December 30, 2010 at First People’s Hospital, Shanghai Jiao Tong University were included in this study. The number of patients meets the requirement of the estimated size for two-sample comparison of survivor functions (Log-rank test, Freedman method, α = 0.05, power = 0.80). CRC was diagnosed by morphological features and immunohistochemical staining. Information about lymph node and distant metastasis and disease-related death were obtained from medical records or telephone interview. 28 peritumoral tissues were collected from the 73 CRC patients during the same time period. All procedures were done ethically with pre-approval from the Ethics Committee for Human Studies, Shanghai Jiao Tong University.

Among the 73 patients, 39 were male and 34 were female with a mean age of 70 years and a range of 37 to 93 years. Staging was performed according to the TNM system recommended by the American Joint Committee on Cancer [20]. Five cases were stage I, 34 were stage II, 30 were stage III, and 4 were stage IV. Lymph node metastasis was found in 36 cases, and distant metastasis was found in 4 cases. 27 patients died during the follow-up. The average follow-up period was 41.137 months with a maximum of 71 months and a median of 48 months. Clinical information about the 73 CRC samples is described in detail in Table 1.

**Table 1:** Summary of clinical characteristics in 73 patients of colon cancer

Characteristics		Patient no. (%)
Sex	Male	39(53.42)
	Female	34(46.58)
Age	Mean	70 ± 12.14
	Median	72
	Range	37-93
	≤70 y	30(41.10)
	>70 y	43(58.90)
TNM stage	Stage I	5(6.85)
	Stage II	34(46.58)
	Stage III	30(41.10)
	Stage IV	4(5.48)
Lymph nodes	Negative	37(50.68)
	Positive	36(49.32)
Metastasis	Negative	69(94.52)
	Positive	4(5.48)

### Tissue microarrays

Tissue microarrays (TMAs) were created from formalin-fixed and paraffin-embedded tissue blocks at the Department of Pathology, First People’s Hospital, Shanghai Jiao Tong University. The slides of 73 patients were reviewed by an experienced pathologist and the representative area of each tumor was marked on the slide. All cases were adenocarcinoma with different degrees of differentiation.

### Immunohistochemistry staining and scoring

The tissue slides were deparaffinized, and antigen retrieval was performed by immersing the slides into boiling EDTA-Tris buffer (pH 8.2) for 4 min. After incubation with 3 % H_2_O_2_ for 15 min, slides were incubated with primary antibody overnight (4 °C), followed by Gtvision III detection system (Gene Tech, Shanghai, China) as secondary antibody for 30 min at room temperature. DAB (3,3-diaminobenzidine) was used to visualize positive immune reaction. Nuclei were counterstained with hematoxylin. The primary antibodies included CC3 (Cell Signaling Technology, 1:300 dilution), HMGB1 (Cell Signaling Technology, 1:400 dilution), RAGE (Abcam, 1:100 dilution), and Ki67 (Epitomics, 1:1000 dilution) antibody.

HMGB1 and RAGE immunoreactivity was scored and put into 3 categories based on staining extent and intensity according to a previously described protocol [14, 21]: negative expression was assigned when almost all cells showed no immunoreactivity or uncertain weak staining, low expression was assigned when less than 25 % of tumor cells showed weak to moderate immunoreactivity, high expression was assigned when more than 25 % of tumor cells showed moderate to intense immunoreactivity. CC3 was scored and put into 3 categories based on staining extent (negative for no staining or uncertain staining, ≤10 % positive staining for low expression, >10 % positive staining for high expression) [3]. Ki67 index was assessed by estimation of the percentage of positive tumor cells (low expression for 0 % to 40 % positive staining, high expression for 41 % to 100 % positive staining) [22, 23]. The representative images are provided in Additional file 1: Figure S1.

### Statistical analysis

Categorical variables were assessed using the *χ*^2^ test and Fisher exact test. The correlation between HMGB1 and CC3, RAGE, or Ki67 was examined using Spearman’s rank method. The cumulative survival time was obtained using the Kaplan-Meier method and compared by the log-rank test. Cox proportional hazards model was used for multivariate analysis. All probabilities were 2-tailed. A *p* <0.05 was considered statistically significant. All statistical analyses were performed using Stata 12.0 software for Windows (StataCorp, Texas, USA).

## Results

### Dying cells stimulate tumor cell proliferation

**Fig. 1:**
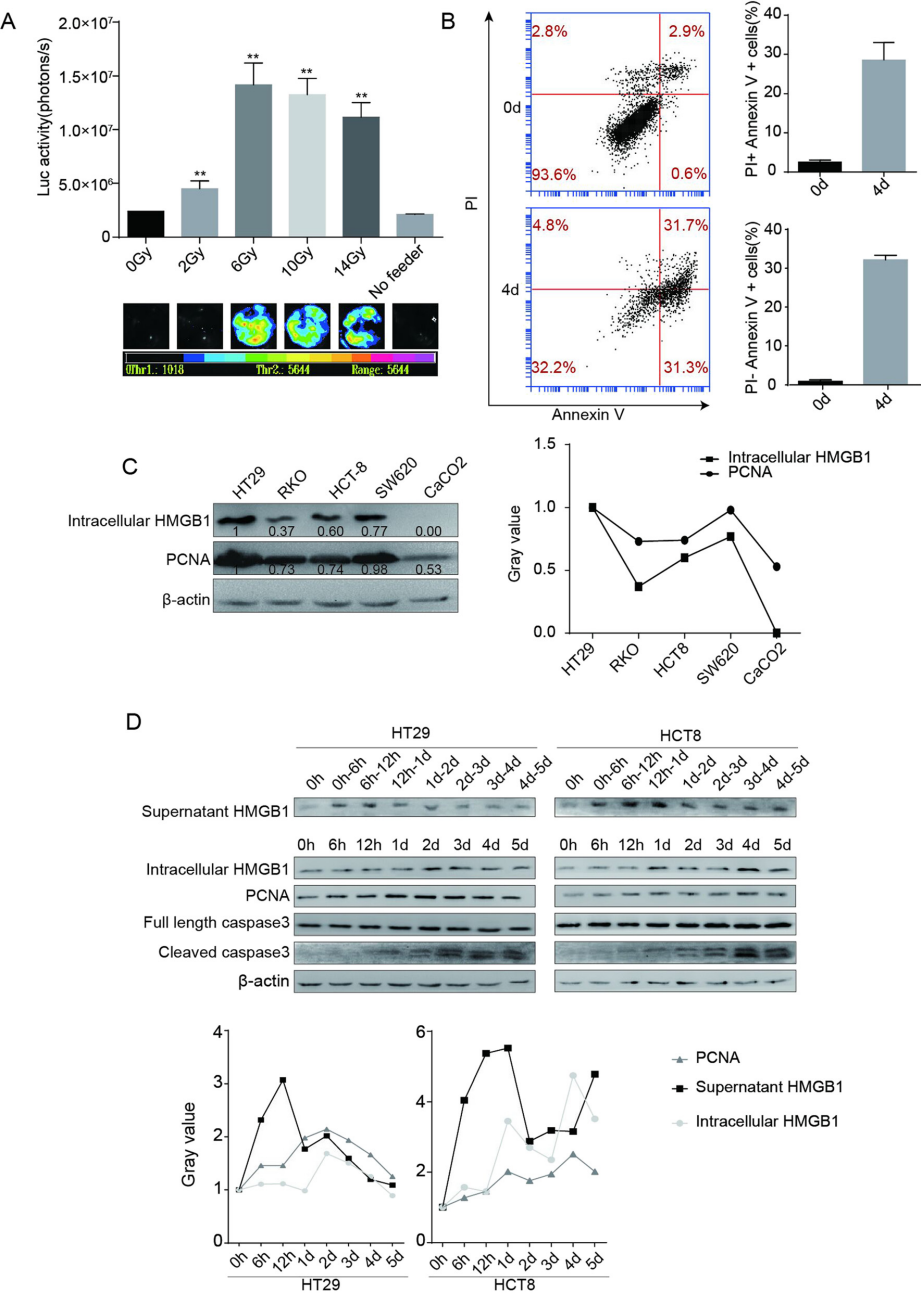
HMGB1 stimulated cell proliferation after irradiation. **(a)** The irradiated HT29 cells (feeder cells) significantly stimulated HT29 Fluc labeled cells growth compared to non-irradiated HT29 feeder cells or reporter alone cells (No feeder). **p <0.01. **(b)** Cell death detection by flow cytometry. **(c)** Western blot of correlated expressions of HMGB1 and PCNA protein in colon cancer cells. Relative intensity changes of HMGB1 and PCNA were analyzed through gray value using software ImageJ. Positive correlation between HMGB1 expression and proliferative marker was observed in colon cell lines. **(d)** Western blot of HMGB1, PCNA, and caspases-3 levels in HT29 and HCT8 cells. Cells were irradiated at 10 Gy. The supernatant HMGB1 was elevated significantly in both cell lines, while there was no obvious change of intracellular HMGB1. PCNA and increased caspases-3 cleavage were also observed after irradiation

The co-cultured cell model [3] was used to investigate the role of dying cells in tumor cell proliferation. Unlabeled HT29 cells (feeder) were irradiated at various doses (0, 2, 6, 10, and 14 Gy) and then co-cultured with Fluc labeled living HT29 cells (reporter). Bioluminescence signals released by reporter cells were used to gauge the extent of proliferation of living tumor cells. As shown in Fig. 1a, all irradiated feeders, especially those that were irradiated with a dose higher than 6Gy, could stimulate living tumor cell proliferation, which was characterized by a significant increase in luciferase activity of reporter cells. This observation suggests that, after irradiation, dying cells can stimulate tumor cell growth. Although apoptotic cells can promote tumor growth and proliferation of surviving cells after tumor irradiation [3], this study showed that the percentage of apoptosis in cell death modalities was not very high. In contrast, necrotic cells were also observed (Fig. 1b).

### Secretory HMGB1 coupled with increased CC3 after irradiation *in vitro*

Extracellular HMGB1 is thought to be an optimal “necrosis marker” [24, 25] and PCNA is a well-known proliferative marker [26]. Western blot showed that the signal intensities of the two proteins were higher or lower in colon cancer cell lines (Fig. 1c), indicating the positive correlation of HMGB1 with a proliferative marker.

To explore the role of HMGB1 in tumor cell proliferation, HT29 and HCT8 cells in exponential growth phase were irradiated for 10Gy, and secretory HMGB1 in cell culture supernatant and intracellular HMGB1 in cell lysate was detected simultaneously by Western blot. We observed that secretory HMGB1 increased at 6 h after radiotherapy. Expression of the proliferative marker PCNA was also found to increase rapidly after radiotherapy. CC3 was significantly increased (Fig. 1d). These findings suggest that the increase in HMGB1 release was accompanied by an increase in CC3 protein level.

### The clinical significance of HMGB1, CC3, RAGE, and Ki67 in colorectal cancer patients

HMGB1, RAGE, CC3 and Ki67 proteins were detected by immunohistochemistry. CC3 positive staining was mainly localized in the cytoplasm and some at the membrane, while HMGB1, RAGE and Ki67 were localized in the nucleus (Additional file 1: Figure S1). Positive HMGB1, CC3, and Ki67 expression was significantly higher in tumor tissues than in peritumoral tissues (*p* <0.01, Table 2). The high CC3 levels positively correlated with high TNM staging and distant metastasis (Table 3). Positive RAGE expression correlated with higher TNM staging and lymph node metastasis. Ki67 expression was found be associated with lymph node metastasis. In contrast, HMGB1 was unexpectedly found to be associated with neither TNM staging nor metastasis.

**Table 2:** Comparison of HMGB1, CC3 and Ki67 expressions in colorectal cancer with peritumor tissues

		Tumor tissue (n)	Peritumor tissue (n)	*p* value
HMGB1	Negative	12	20	0.000*
	low	29	8	
	high	32	0	
CC3	Negative	18	26	0.000*
	low	35	2	
	high	20	0	
Ki67	low	38	24	0.003*
	high	35	4	

*,statistically significant

**Table 3:** Correlation of clinicopathologic parameters with HMGB1, CC3, Ki6 and RAGE expressions

	HMGB1 + (%)	*p*	CC3 + (%)	*p*	Ki67 + (%)	*p*	RAGE+(%)	*p*
Sex		0.964		0.081		0.121		0.524
Male	17(43.59)		14(35.90)		22(56.41)		26(66.67)	
female	15(44.12)		6(17.65)		13(38.24)		25(73.53)	
Age		0.684		0.138		0.769		0.589
≤70 y	14(46.67)		11(36.67)		15(50)		22(73.33)	
>70 y	18(41.86)		9(20.93)		20(46.51)		29(67.44)	
TNM stage		0.496		0.007*		0.321		0.03*
Stage I	3(60)		2(40)		2(40)		1(20)	
Stage II	14(41.18)		7(20.59)		13(38.24)		22(64.71)	
Stage III	12(40)		7(20.59)		17(56.67)		25(83.33)	
Stage IV	3(75)		4(100)		3(75)		3(75)	
Lymphnodes		0.565		0.551		0.007*		0.050*
Negative	15(40.54)		9(24.32)		12(32.43)		22(59.46)	
Positive	17(47.22)		11(30.56)		23(63.89)		29(80.56)	
Metastasis		0.196		0.001*		0.265		0.818
Negative	29(42.03)		16(23.19)		32(46.38)		48(69.57)	
Positive	3(75)		4(100)		3(75)		3(75)	

+High level expression of proteins

*p value ≤0.05. The difference of protein expressions between clinicopathological parameters was statistically significant

**Fig. 2:**
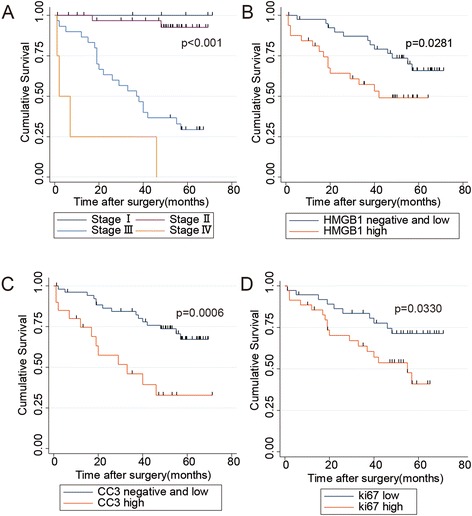
Kaplan-Meier survival analysis in 73 cases of patients with colorectal cancer. The patients with higher stage **a**, HMGB1 **b**, CC3 **c**, Ki67 **d**, expression were associated with shorter overall survival time

The Kaplan-Meier survival analysis revealed that TNM staging was clearly correlated with overall survival in CRC (Fig. 2a). Interestedly, high HMGB1 expression was significantly associated with poor prognosis. In addition, positive CC3 and Ki67 expression was also significantly correlated with shorter survival time in CRC patients (*p* = 0.0281, *p* = 0.0006, *p* = 0.033, respectively; Fig. 2b, c, d, Table 4). Multivariate analysis using Cox proportional hazards model showed that TNM staging and HMGB1 (*p* = 0.000 and *p* = 0.008, respectively), but not CC3 and Ki67 (*p* = 0.998 and *p* = 0.347, respectively) were independent prognostic factors in CRC patients (Table 4).

**Table 4:** Univariate and multivariate survival analysis of overall survival

Variables	Univariate analysis	Multivariate analysis		
	*p*	*p*	RR	95 % CI
Sex (female vs male)	0.5873			
Age (≤70 y vs >70y)	0.0962			
TNM stage	0.0000*	0.000*	8.19	3.5370-19.0053
Lymph nodes (+ vs -)	0.0000*			
Metastasis (+ vs–)	0.0000*			
CC3 (high expression vs otherwise)	0.0006*	0.998	1.00	0.3627-2.7655
HMGB1 (high expression vs otherwise)	0.0281*	0.008*	3.55	1.3972-9.0147
RAGE (high expression vs otherwise)	0.3119			
Ki67 (high expression vs otherwise)	0.0330*	0.347	1.50	0.6451-3.4806

CI: confidence interval, RR: risk ratio. *statistically significant

**Fig. 3:**
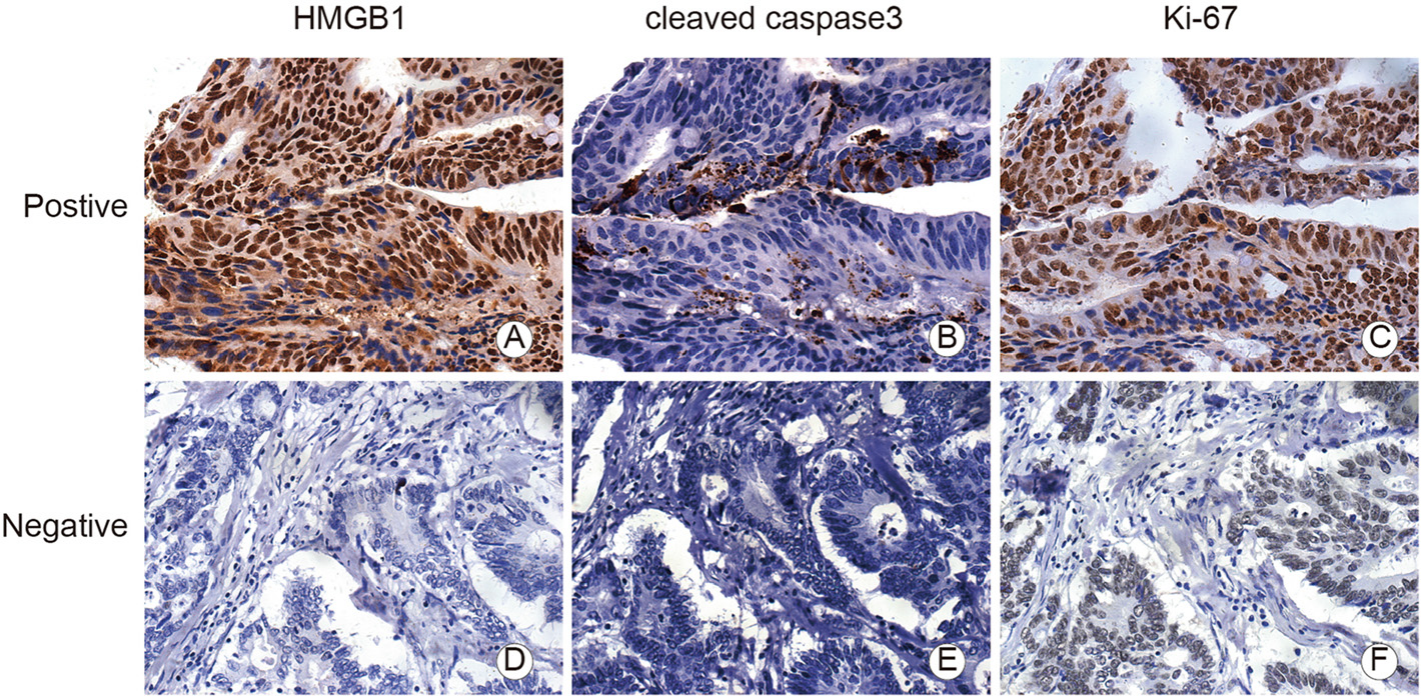
HMGB1, CC3, and Ki67 expression in colorectal cancer tissues in serial sectioning slides. (**a**, **b** and **c**) represented the positive immunohistochemical staining of HMGB1, CC3, and Ki67, respectively, while **d**, **e** and **f** represented the negative staining of the three proteins

Spearman’s rank correlation analysis further showed that HMGB1 positively correlated with CC3 and Ki67 level (correlation coefficient 0.5393, 0.3814, respectively; *p* <0.001, Table 5). Fig. 3 showed a coordinated expression of HMGB1, CC3, and Ki67 that they were either all positive or all negative/low expression in the same visual field.

**Table 5:** Relationship between HMGB1 and other protein expression in 73 cases of colorectal cancer

		Total no. (%)		HMGB1, n		ρ(rho)	*p* value
			Negative	Low	High		
CC3	Negative	12(16.44)	8	7	3	0.5393	0.000*
	low	29(39.73)	3	20	12		
	high	32(43.84)	1	2	17		
RAGE	Negative	5(6.85)	3	0	2	0.0047	0.9683
	low	17(23.29)	2	6	9		
	high	51(69.86)	7	23	21		
Ki67	low	38(52.05)	11	16	11	0.3814	0.0009*
	high	35(47.95)	1	13	21		

ρ(rho): Spearman correlation coefficient

*Correlation is statistically significant at 0.05 level (2 tailed)

## Discussion

The purpose of tumor therapy is to induce tumor cell death. However, tumor cell death is not the final step in overcoming cancer. There is increasing evidence showing that dying or dead tumor cells can actually promote tumor development through many mechanisms. Therefore, it is necessary to investigate the relationship between death and growth of tumor cells. Our previous studies have demonstrated that apoptotic tumor cells can stimulate the proliferation of residual living tumor cells through activating caspase-3 and its downstream effectors [2, 3]. However, apoptosis is not the only death modality for irradiated tumor cells. Necrosis is also present after irradiation. The mechanism behind dying cells stimulating the growth of living tumor cells might be rather complex. In this study, we investigated the role of apoptotic and necrotic cells in tumor progression, and found that, after irradiation, the dying colorectal cancer cells were composed of both apoptotic and necrotic cells. After irradiation, we were able to detect secreted HMGB1 before caspase-3 cleavage. Interestingly, a cell proliferation marker, PCNA, was found to be increased after irradiation, and its expression paralleled the increased expression of HMGB1 in the medium after irradiation. In addition, we also examined clinical colorectal cancer samples and found that HMGB1 is also a marker for poor prognosis. Therefore, this study may uncover a new mechanism that besides the caspase-3 from apoptotic cells, some other signal molecules, such as HMGB1 released from the necrotic cells, may also exert a role in the proliferation of living colorectal cancer cells.

Because active HMGB1 can be released from dead, dying, and injured cells, it could be a good marker for necrosis. Obviously, this concept is inaccurate with regards to current research [7, 8, 27]. This study showed that the concentrations of HMGB1 in the culture medium of colorectal cancer cells and cleaved caspase-3 in the colorectal cancer cells were increased after radiation, which parallels the increase in PCNA, a well-known proliferative marker. This finding supports the idea that there is a balance between cell death and proliferation in irradiated cells. Although nuclear HMGB1 acts as a DNA chaperone, the extracellular HMGB1 is widely thought of as a signaling molecule and involved in a variety of physical and pathological processes, such as inflammation, cell proliferation and differentiation, and tissue regeneration [6, 8, 27]. It is therefore not difficult to believe that HMGB1 acts as a proliferation factor that is involved in the dying-cell-stimulated proliferation of living tumor cells in this study.

Extracellular HMGB1 acts through binding to several cell surface receptors, including RAGE [8, 9]. The binding of HMGB1 to RAGE is thought to stimulate proliferation and differentiation of cancer cells [10]. Unfortunately, the dynamic changes in the expression of RAGE in the proliferating living colorectal cancer cells were not evident. However, in this study, the positive RAGE expression correlated with higher TNM staging and lymph node metastasis in colorectal cancer patients. The TNM system is one of the most widely used cancer staging systems that categorizes the stage of a solid cancer based on the size and/or extent of the primary tumor (T), the amount of spread to lymph nodes (N), and the presence of metastasis (M) [20]. In general, higher TNM staging represents rapid tumor progression and highly aggressive tumors. The positive correlation between RAGE expression and TNM stage suggests that RAGE could possibly be a factor that influences tumor prognosis. This is also consistent with a previous report [14].

In addition, this study confirmed the roles of CC3 in the proliferation of tumor cells as well as its association with poor prognosis in colorectal cancer patients [3]. The role of proliferative marker Ki67 in colorectal cancer patients was also confirmed and consistent with PCNA expression in tumor cells studied *in vitro* as well as a previous study in CRC patients [22]. This suggests that the data from the current case series are suitable for analysis of the clinical significance of HMGB1. Although HMGB1 showed no association with TNM staging and metastasis, it did correlate with poor prognosis (*p* = 0.0281) and is a predictive marker for poor prognosis according to multivariate analysis. Based on the histological study and *in vitro* study, we propose that both apoptosis and necrosis play an important role in tumor progression, and both CC3 and HMGB1 are predictive markers of poor prognosis in colorectal cancer patients.

Repopulation describes the phenomenon that surviving tumor cells after radiotherapy reestablish and form a new tumor [28]. However, the underlying mechanism has not been fully elucidated. It could be caused by proliferation from lightly injured tumor cells as a result of heterogeneous reaction to radiation or living tumor cells that migrated beyond the radiated field. The results from our study suggested that apoptotic and necrotic cells could stimulate proliferation of the living tumor cells. The HMGB1 released from dying cells could be a new marker to predict the possibility of tumor repopulation. This may also represent a new mechanism of tumor repopulation.

In conclusion, repopulation is one of the main obstacles hindering successful cancer treatment. The apoptotic or necrotic cells can stimulate growth of living tumor cells and subsequently mediate the relapse of colorectal cancer after radiotherapy. The increased expression of the key factors of apoptosis or necrosis such as CC3 and HMGB1 in tumor could be a new marker for poor prognosis in colorectal cancer.

## Additional file

Additional file 1: Figure S1. The representative immunohistochemical staining of different protein expressions (original magnification × 400). a), b) and c) for negative, low, and high CC3 expression, respectively; d), e) and f) for negative, low, and high HMGB1 expression, respectively; g), h) and i) for negative, low, and high RAGE expression, respectively; j) and k) for low and high Ki67 expression, respectively.
