# Supplementary figures and images for: Increased HMGB1 and cleaved caspase-3 stimulate the proliferation of tumor cells and are correlated with the poor prognosis in colorectal cancer
Source: J Exp Clin Cancer Res. 2015 May 20;34(1):51. doi: 10.1186/s13046-015-0166-1 (PMC4446854; doi:10.1186/s13046-015-0166-1)

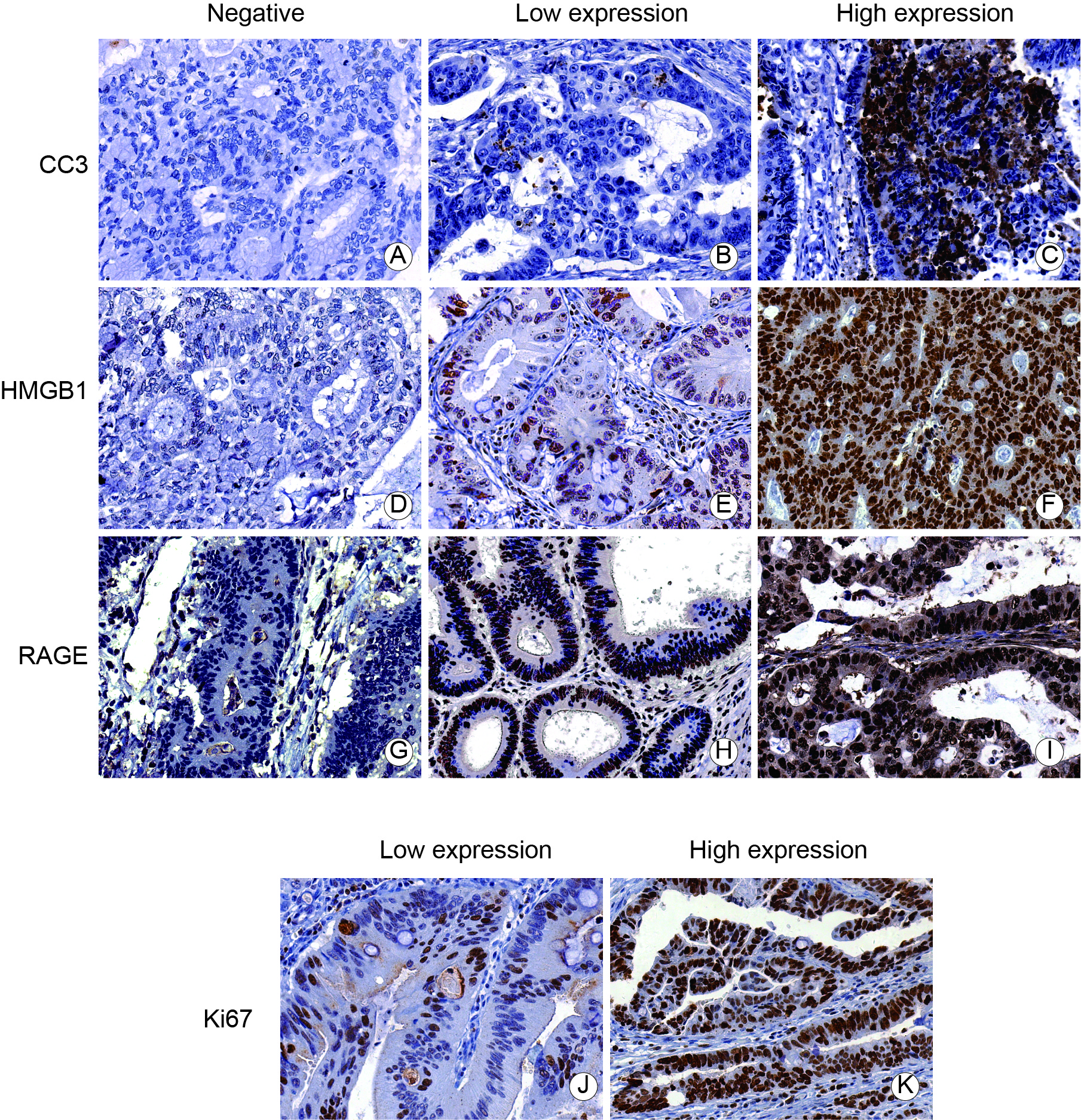

Supplement: Supplementary file 1 — The representative immunohistochemical staining of different protein expressions (original magnification × 400). a), b) and c) for negative, low, and high CC3 expression, respectively; d), e) and f) for negative, low, and high HMGB1 expression, respectively; g), h) and i) for negative, low, and high RAGE expression, respectively; j) and k) for low and high Ki67 expression, respectively. [file 13046_2015_166_MOESM1_ESM.jpg]
